# Surface Acoustic Wave Propagation of GaN/Sapphire Integrated with a Gold Guiding Layer

**DOI:** 10.3390/s23052464

**Published:** 2023-02-23

**Authors:** Muhammad Musoddiq Jaafar, Mohd Farhanulhakim Mohd Razip Wee, Hoang-Tan-Ngoc Nguyen, Le Trung Hieu, Rahul Rai, Ashish Kumar Sahoo, Chang Fu Dee, Edward Yi Chang, Burhanuddin Yeop Majlis, Clarence Augustine TH Tee

**Affiliations:** 1 College of Physics and Electrical Information Engineering, Zhejiang Normal University, Jinhua 321017, China; 2 Institute of Microengineering and Nanoelectronics, Universiti Kebangsaan Malaysia, Bangi 43600, Selangor, Malaysia; 3 International College of Semiconductor Technology, National Chiao Tung University, University Road, Hsinchu 30010, Taiwan; 4 Department of Materials Science and Engineering, National Chiao Tung University, University Road, Hsinchu 30010, Taiwan; 5 Department of Electronics Engineering, National Chiao Tung University, University Road, Hsinchu 30010, Taiwan

**Keywords:** gallium nitride, surface acoustic waves, Sezawa waves

## Abstract

Gallium nitride (GaN), widely known as a wide bandgap semiconductor material, has been mostly employed in high power devices, light emitting diodes (LED), and optoelectronic applications. However, it could be exploited differently due to its piezoelectric properties, such as its higher SAW velocity and strong electromechanical coupling. In this study, we investigated the affect of the presence of a guiding layer made from titanium/gold on the surface acoustic wave propagation of the GaN/sapphire substrate. By fixing the minimum thickness of the guiding layer at 200 nm, we could observe a slight frequency shift compared to the sample without a guiding layer, with the presence of different types of surface mode waves (Rayleigh and Sezawa). This thin guiding layer could be efficient in transforming the propagation modes, acting as a sensing layer for the binding of biomolecules to the gold layer, and influencing the output signal in terms of frequency or velocity. The proposed GaN/sapphire device integrated with a guiding layer could possibly be used as a biosensor and in wireless telecommunication applications.

## 1. Introduction

Gallium nitride (GaN) has been widely used in high-speed devices [1], high-power devices [2], and optoelectronic devices [3] due to its superior wide bandgap [4], high voltage and current capacity [5], high critical breakdown electric field, and significantly high electron mobility properties [4]. On the other hand, the piezoelectric characteristics of GaN has been acknowledged in many exciting potential applications in surface acoustic wave (SAW) devices [6,7,8]. In more profound discussions, GaN SAW devices are promising candidates to substitute traditional SAW substrates such as lithium tantalate, quartz, etc. This is due to GaN SAW devices’ ability to operate at a very high operating frequency, their ability to work in harsh environments, their potential for direct unification with optoelectronics devices, their higher SAW velocities (3704 m/s), their small temperature coefficient delay, and their strong electromechanical coupling (K${}_{2}$eff = 2.0) [9].

Nowadays, GaN-based devices consist of several layers deposited on a bulky substrate such as sapphire or silicon, realized by the growth in high-quality GaN epilayers on sapphire substrates using the breakthrough metal organic chemical vapour deposition (MOCVD) technique [10], which could reduce costs. This heterostructure could be employed directly as an SAW device without any modification to improve its compatibility and integration with the current CMOS process in a single chip [11,12]. Furthermore, the heterostructure configuration of the GaN layer and sapphire substrate for SAW devices could produce a wide spectrum of acoustic modes, which arise from the mismatch of elastic properties between the layer and the substrate [13].

Currently, SAW devices are fabricated with bulk piezoelectric materials and are commonly used to convert AC electrical signals into a mechanical vibration. Piezoelectric materials, such as lithium niobate (LiNBO3), PZT, and quartz, are preferred by industry due to their low loss properties and high SAW velocities. However, these bulk materials operate at frequencies lower than 2 GHz, which limits their performance, especially in high frequency telecommunication applications. Nonetheless, various heterostructures could be employed to achieve higher operating frequencies by exploiting higher order surface modes (Sezawa) other than Rayleigh modes, without any requirement for advanced nanolithography techniques in order to obtain a nanoscale structure. Typically, the first surface mode, usually denoted as Rayleigh-type or typically called a Rayleigh mode, yields a transverse and longitudinal displacement component which occurs at the top surface and decreases exponentially in amplitude as the distance from the surface increases. Recently, surface modes known as Sezawa modes, which appear in layered structures such as GaN/sapphire and AlN/diamond, have led to higher velocities compared to the fundamental Rayleigh mode, using a similar finger geometry and spacing in the interdigitated transducers (IDT), as demonstrated previously by Muller et al. [9]. This Sezawa mode appears only if the value of bulk transverse velocity in the substrate is higher than the transverse velocity in the over layer (GaN) to create a “slow on fast” structure. It is also restricted to certain values of the normalized thickness, with the propagation mostly found in the over layer and polarized in the sagittal plane.

**Figure 1:**
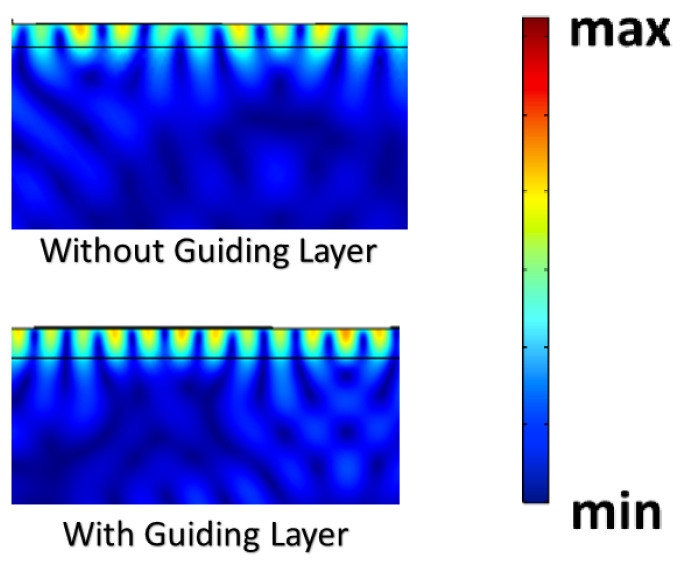
The **top** image shows a simulation of GaN on top of sapphire without a guiding layer. The **bottom** image is a simulation of GaN on top of sapphire with a guiding layer. The scale bar showed the higher energy to lower energy density.

Meanwhile, the presence of guiding layers has also been demonstrated to be useful in improving sensor performance. The comparison in Figure 1 shows the guiding layer will improve the energy density at the surface and will yield a better performance of the sensor, since the presence of guiding layers concentrates the energy of the wave onto the surface, which reduces its dissipation into the bulk of the device, thus rendering a higher sensitivity to surface perturbations. We expect a shift to lower frequency will occur with the addition of a guiding layer, and hence a reduction in the phase velocity, which is in agreement with the Sauerbrey equation from perturbation theory [14].

In this study, we proposed higher operating frequency SAW devices by exploiting the confined modes which appear in the so called “slow on fast” structures such as, in our case, GaN/sapphire, where the acoustic velocity in the thin layer is smaller than in the substrate. The confined mode (Sezawa mode) has a higher velocity than the fundamental Rayleigh mode in order to obtain a higher resonance frequency. Through both experimental and simulation studies, it was found that the addition of a thin guiding layer made from gold did not disturb the wave propagation of all surface modes in GaN/sapphire in terms of velocity and frequency and proved the advantages of the Sezawa mode (instead of the fundamental Rayleigh mode) in applications such as Sezawa-based biosensors with the guiding layer acting as a binding spot for the targeted biomolecules. Our proposed GaN/sapphire heterostructure not only produces the traditional Rayleigh mode, but we also report the presence of additional modes which could be further exploited for future development and integration of various structures (filters, oscillators, and sensors) for smart communication-processing circuit technology.

## 2. Methodology

### 2.1. Experimental Methods

**Figure 2:**
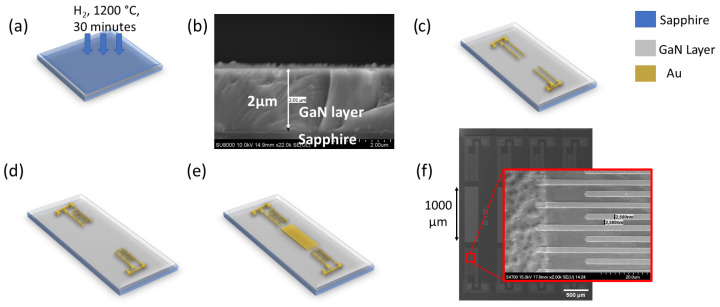
Thermal cleaning of the sapphire substrate by using gaseous H2 at 1200 °C for 30 min (**a**), cross-section of GaN growth on top of sapphire with an AlN buffer (**b**), coplanar wavepad deposited on top of the GaN layer (**c**), variation in IDTs: 2.5 µm-, 5 µm-, and 7.5 µm-width are deposited (**d**,**e**), a thin film of Au/Ti (200 nm/20 nm) is deposited, which acts as a guiding layer, (**f**) final SEM structure of the SAW devices.

The gallium nitride epitaxial layers on the sapphire substrate were grown using a Thomas Swan 1200 MOCVD reactor. The growing method makes use of trimethylgallium (TMGa), trimethylallium (TMAl), and ammonia (NH${}_{3}$) as the precursors for Ga, Al, and N sources, while hydrogen gas (H${}_{2}$) and nitrogen gas (N${}_{2}$) were utilised as the carrier gases for the growth process. Before the growth of the AlN buffer, a thermal cleaning process of two-inch sapphire substrates was performed by using gaseous H${}_{2}$ at 1200 °C for 30 min, as shown in Figure 2a. Then, a nucleation layer of AlN buffer of 125 nm thickness was deposited on the sapphire substrate under the growth conditions of 1200 °C and a V/III ratio of 395. Afterwards, a 2 µm-thick GaN layer was grown under the conditions of 1125 °C and a V/III ratio of 433, which is shown in the cross-section SEM image in Figure 2b. The AlN buffer layers produce the best crystalline quality with low tensile stress of the GaN layer.

After the fabrication of the GaN/sapphire device was completed, the substrate was washed using acetone and isopropyl alcohol for 5 min in a sonication bath for each cleaning chemical. Then, the photolithography process to fabricate a coplanar wave pad was performed by coating the AZ5214E photoresist with a spin speed of 400 rpm for 30 s. Subsequently, the photoresist was heated to 90 °C for 90 s to remove the coating solvent and improve adhesion to the substrate. Next, the coplanar waveguide pattern was created by exposing the photoresist to light. An image reversal process was performed to ensure the unexposed photoresist was removed throughout the developing process. Then, metallization of Ti/Al/Ni/Au with thicknesses of 20 nm, 100 nm, 25 nm, and 100 nm, respectively, was performed, as illustrated in Figure 2c. Afterwards, the same lithography step was repeated with an IDT mask pattern of 2.5 µm, 5 µm, and 7.5 µm IDT width with metallization of Ni/Au (Schottky contact) with thicknesses of 20 nm and 200 nm, respectively, as in Figure 2d.

Subsequently, a 1000 µm-long guiding layer was deposited with the same lithography process and similar IDT metallization layers (20 nm and 200 nm layers of Ti/Au, respectively) for each width, as in Figure 2e. A top view of the completed devices with a 2.5 µm IDT width is shown in Figure 2d with a set of transmission IDTs and receiving IDTs. The crystalline quality of the GaN layer was examined using X-ray diffraction (XRD) Bede D1 HR-XRD, Bede Scientific Instruments, Colorado, United States and the surface morphology was investigated by using atomic force microscopy (AFM) (Dimension Edge, Bruker, Germany). Subsequently, the RF frequency characteristics were analyzed using the E8361C Network Analyzer, Keysight Technologies, California, United States.

### 2.2. Theoretical Modelling

**Figure 3:**
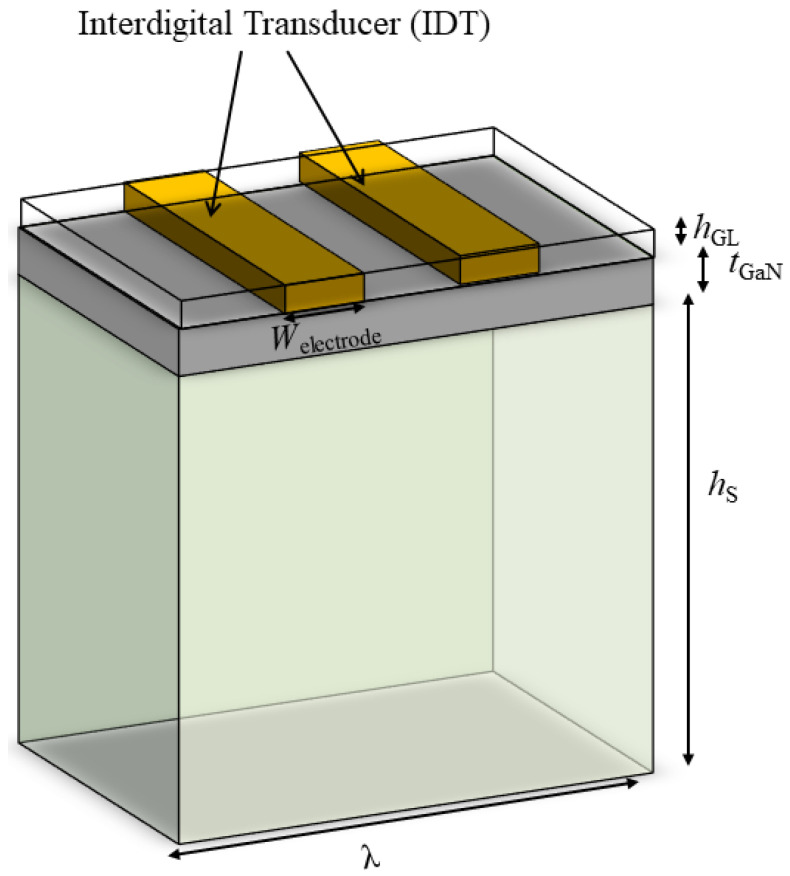
Schematic diagram of the 3D model of a unit cell of a GaN/sapphire device used in the FEM simulation method.

The simulation of a unit cell of GaN/sapphire is based on Figure 3, which is replicated from the final structure of GaN devices in the previous section, in order to simulate the frequency response and to identify the propagation modes of our structure. For the simulation analysis, the finite elemental method (FEM) based on a three-dimensional (3D) periodic model was used, varying the width, *w*, of the electrode (2.5, 5, and 7.5 µm), while keeping the wavelength, λ (10 µm), the sapphire thickness, $h_{s}$ (30 µm), the GaN thickness, $t_{GaN}$ (2 µm), the guiding layer thickness, $h_{GL}$ (200 nm), and the electrode thickness (200 nm) constant throughout the entire simulation of the unit cell.

Concurrently, the top boundary of the model was not a fixed constraint, while the bottom boundary was set to be a fixed constraint. Moreover, the height of the model was set to be a few times longer than the wavelength, which is sufficiently large for the surface wave to be completely attenuated before it reaches the bottom boundary. We implemented the Bloch–Floquet periodic condition on the lateral faces on the left and right sides of the structure for simplification and to allow for unit cell assumption. In the meantime, the material parameters, such as the elasticity constant and the piezoelectric coefficients for GaN and sapphire, were taken from ref. [13], while other material parameters were taken directly from the COMSOL library.

Next, an FEM analysis was conducted in which the HAR electrode on the GaN/sapphire heterostructure layer facilitates the excitation of Sezawa surface mode simulation. Differential equations were engaged to determine the mechanical, structural, and electrical problems on the model complexity (the geometry model, material properties, and the boundary conditions). For the piezoelectric devices, the mechanical equation of motion governs the SAW propagation, while Maxwell’s equation was used for electrical behavior. Furthermore, the FEM also pairs the electrical field, electrical displacement, stress, and strain of the GaN piezoelectric layers. In this simulation model, COMSOL determined the structural and electrical equations of the GaN piezoelectric layers, which was applied for the substrate and guiding layers.

As for the metallic layers of Au, the electrical equation was not applied, as the electrical conductivity is few orders of magnitude higher than the piezoelectric GaN layers, which appear as equipotential regions that only allow a minimal amount of conducting current across them. Instead, an electrical boundary condition was applied as the alternating electrical potential though the electrode by adjusting a positive potential of 2 V with zero surface accumulation, which correlates to an open circuit. The applied alternating electrical potential excited the surface waves and introduced mechanical deformations into the piezoelectric layers by implying inverse piezoelectric phenomena. Since the metal electrode was supposed to be perfectly conducting, the electrical equations were not solved in the electrode. Meanwhile, the piezoelectric domain of GaN behaves as an insulator with a conductivity which is several orders of magnitude lower than the metallic electrode.

## 3. Results and Discussions

**Figure 4:**
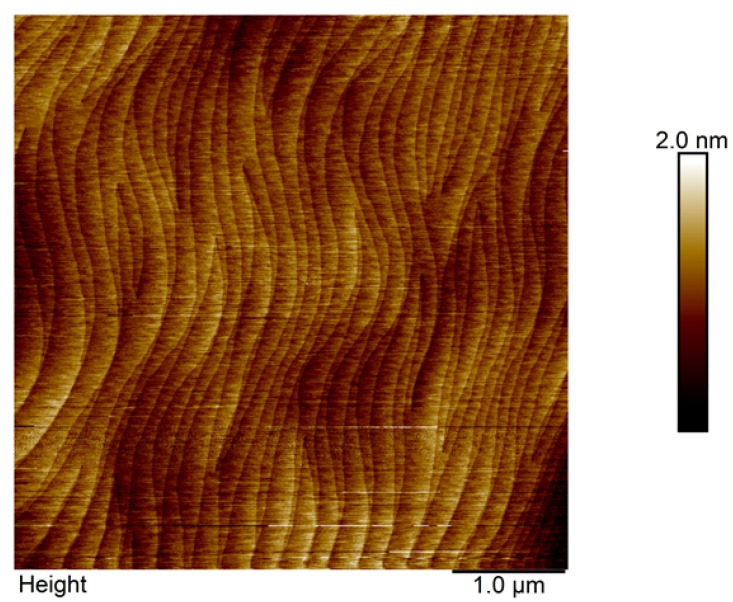
AFM analysis of a 5 × 5 µm scanning area for a 2 µm-thick GaN epitaxial layer on top of a sapphire substrate.

**Figure 5:**
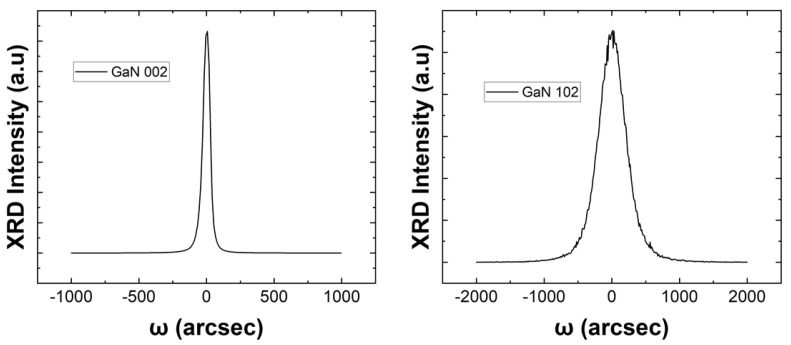
The symmetric (002) and asymmetric (102) rocking curves of GaN grown on the sapphire substrate with am AlN buffer by using the MOCVD method.

Various studies have shown that the growth of the AlN buffer could reduce the lattice mismatch stress and thermal mismatch stress between the substrate and the GaN epitaxial layers [15]. This leads to high quality GaN epitaxial layers. The benefit of the AlN buffer is demonstrated in this work, as the AFM image of the GaN epitaxial layer, shown in Figure 4, shows a fairly smooth GaN epitaxial layer with a lower density of etch pits and without any large waves and grains, which is comparable with the work of Park et al. [15]. Furthermore, analysis of a 5 × 5 µm scanned area showed a very small root mean square value, which was 0.212 nm. It exhibits a definitive smooth area that presents a clear atomic pattern, as seen in the figure. The study of the XRD rocking curves of symmetric (002) and asymmetric (102) planes was performed at the centre of the wafer to evaluate the GaN epitaxial layer’s crystal quality, which is shown in Figure 5. In this study, the full width at half maximum (FWHM) values of the ω-rocking curve for (002) and (102) planes are 62 and 455 arcsec, respectively. This value is comparable to those reported for high quality GaN epitaxial layers on sapphire and CNT/sapphire, which were about 180 to 345 for the (002) plane and 267 to 448 for the (102) plane [16]. The FWHM of the rocking curve mirrors the heterogenous strain and grain size. The rocking curve of the (002) plane corresponds to the C-directional information of the film. Furthermore, the smaller (002) value signifies the C-axial of the GaN epitaxial layer has better relaxation. In our work, we obtained a value of about 62 arcsec, which indicates a higher relaxation.

**Figure 6:**
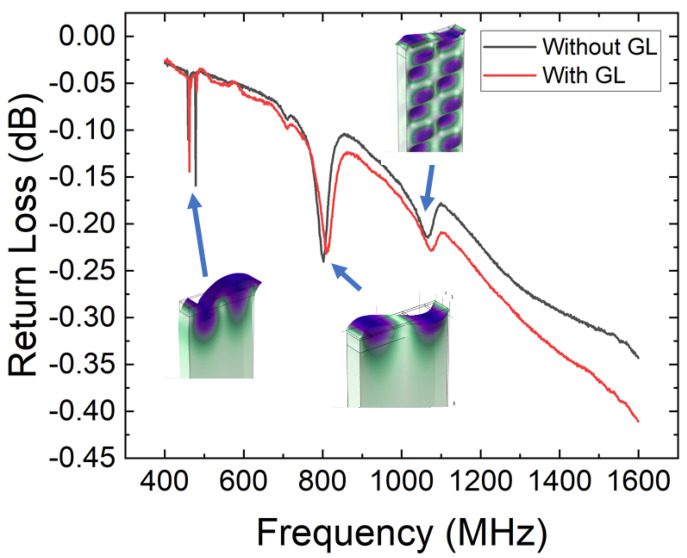
Return loss measurement at room temperature versus frequency under the conditions of without and with a guiding layer for W = 2.5 µm. R, S, and PB denote Rayleigh, Sezawa, and pseudo-bulk, respectively.

Next, the propagation of different acoustic modes in GaN grown on sapphire was examined. As shown in Figure 6, the return loss vs. frequency graph for W = 2.5 µm, with the conditions of with and without a guiding layer (GL), showed an apparent three resonance peak. By using the values of the Rayleigh mode velocity, as well as the values of the transverse velocity in sapphire and GaN, we could estimate that the three resonances correspond to the Rayleigh (R), Sezawa (S), and pseudo-bulk (PB) modes [13]. This is due to the bulk transverse wave of GaN (3941 m/s) [17] being smaller than the bulk transverse wave of the sapphire substrate (5983 m/s) [18]; hence, this structure is considered to be a slow on fast SAW structure, in which Sezawa waves (S) and pseudo-bulk (PB) are likely to be present alongside the traditional Rayleigh mode (R) in the same IDT size [13,18]. As can been seen in Figure 5, for the case with a guiding layer, the lowest frequency peak at 462 MHz corresponds to the Rayleigh mode, while the middle peak at 809 MHz corresponds to the Sezawa mode, and the frequency higher than the state is presented at the frequency of 1.071 GHz and is the sapphire pseudo-bulk.

In the case without a guiding layer, the lowest peak, which corresponds to the Rayleigh mode, is 459 MHz, while the middle peak, which corresponds to the Sezawa mode, is 802 MHz, and the pseudo-bulk is at 1.066 GHz. Furthermore, by minimizing the thickness of the guiding layer to 200 nm, the effect of mass loading appears to be minimized, as there are no apparent frequency shifts between the devices with and without the presence of a guiding layer, see Figure 5. Moreover, an extensive analysis using the FEM method confirmed the presence of three types of propagation mode, as the 3D model of the wave modes appears at a similar location, corresponding to the frequency peaks in the experimental results. For the first mode (R), the displacement was observed to be concentrated in the electrode and inside the GaN layer, as well inside the top sapphire substrate, while the second mode (L) appeared to propagate through a thin layer of GaN and showed a displacement along the whole thickness of the GaN layer. For the third peak (PB), a strong displacement penetration towards the bottom of the sapphire substrate in the wave propagation, which was noted to have a strong contribution from the bulk sapphire, was observed.

The propagation of phase velocity versus the size of IDT (W = 2.5, 5, and 7.5 µm) under the conditions of with or without a guiding layer is presented in Figure 6 For the case of the Rayleigh mode, with the size of W = 2.5 µm, the propagation speed without the presence of a guiding layer is 4590 m/s, while with the presence of a guiding layer, the propagation speed is 4620 m/s. For W = 5 µm, the propagation speed without a guiding layer is increased by 10.3% to 5060 m/s. With the presence of a guiding layer, a similar increase of 10.8% is also observed to about 5120 m/s. For W = 7.5 µm, there was an apparent increase of 19.6% in propagation speed (5490 m/s) for the case without a guiding layer when compared to W = 2.5 µm, while in the case with a guiding layer, an increase of 16.8% is noted when compared with W = 2.5 µm. In the case of a guiding layer, the apparent change in speed is less than that without a guiding layer, this due to the effect of mass loading, which increases the propagation speed alongside an increase in IDT size. With the decrease in guiding layer thickness to about 200 nm, the effect of mass loading for the same IDT sizes of W = 2.5, 5, and 7.5 µm is not as significant, with a mismatch of 0.65∼1.64%. This minimal mismatch is preferred, as the existence of a guiding layer on top of the piezo layer with the same IDT size usually reduced the propagation speed due to the effect of mass loading.

In this slow on fast structure, a Sezawa mode is observed, as it is typically generated at the interface of two solid elastic substrate layers, where a thin layer is on top of a thick layer. For this mode, a faster velocity of about 74.5% to 90.0% is achieved compared to the Rayleigh mode. For W = 2.5 µm, the propagation speed of the Sezawa mode is about 8010 m/s, which is 74.5% higher than the Rayleigh mode for the case without the presence of a guiding layer, while a similar increase of 75.5% (8110 m/s) also occurs in the presence of a guiding layer compared to the Rayleigh mode for the same IDT size. For W = 5.0 µm, the propagation speed of the Sezawa mode compared to the Rayleigh mode showed a significant increase of 83.3% (9280 m/s) without the presence of a guiding layer, while a decrease in propagation speed of 82.8% (9360 m/s) is observed in the presence of a guiding layer. Moreover, when W = 7.5 µm, an increase of 82.5% (10,020 m/s) is observed when compared with the previous Rayleigh mode without the presence of a guiding layer, while an increase of 90% (10,260 m/s) was observed with the presence of a guiding layer.

The shear horizontal wave in this case is very useful in SAW-based liquid environments for sensor applications, especially for biosensing devices, which typically require liquid surroundings. This is because when in contact with a fluid, a low attenuation of the propagation wave is typically observed, in contrast with the complete damping of the traditional Rayleigh wave [19].

Last but not least, the sapphire pseudo-bulk (PB) appears at a frequency above the transonic state. Interestingly, an increase of 33.2% to 38% is observed for W = 2.5 and 5 µm when compared with the velocity of longitudinal Sezawa waves for both cases of with or without the presence of a guiding layer. However, increases in propagation velocities of 133% and 127% are observed for W = 7.5 µm in both the cases of without and with the presence of a guiding layer, respectively. These fast, leaky modes are predominant in longitudinal waves and attenuate as they propagate. The high transmission amplitude in the GaN/sapphire structure, which disperses deep into the substrate, is due to the partial coupling with bulk waves, which is common for longitudinal waves [13].

A recent report has suggested that the introduction of an SAW guiding layer offers a promising and superior detection sensitivity for physical, chemical, and biosensing. It has proven to be an excellent approach for humidity, temperature, stress, force, and viscosity sensors. Moreover, due to the versatility of GaN SAW devices, it offers an extensive wide range of sensor applications, not only gas sensing, but also for chemical analysis in various media. Moreover, without the guiding layer, the substrate would allow leaky SAWs and the energy would be radiated into liquid media, which would lead to a significant loss when in contact with water. Thus, Sezawa SAW devices appear promising for biological detection because of their low signal attenuation in liquid, fast response time, high sensitivity, direct use of raw biological samples for detection, label-free assay, and simple implementation of array systems. Furthermore, a thin guiding layer offers a functionalized surface to provide a basis for the monolayers of active groups to subsequently couple to analyte-specific molecules. The binding of analytes to the immobilized capture molecules will influence the velocity of the SAW and hence the output signal generated by the driving electronics.

**Figure 7:**
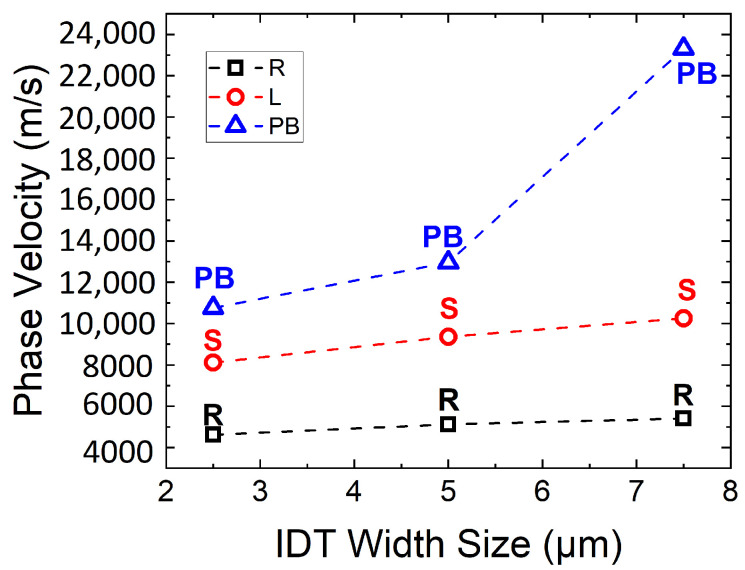
Phase velocity versus IDT width size with the condition of a guiding layer. R is the Rayleigh mode, S is the Sezawa mode, and PB is the pseudo-bulk mode.

The graph in Figure 7 shows the phase velocities for different IDT widths with the presence of a guiding layer. The increase in IDT size directly increased the wavelength of the propagated wave. In the case of the Rayleigh mode, the phase velocity did not appear to increase significantly with the increase in wavelength. However, for the case of the Sezawa mode, the increase in wavelength significantly increases the phase velocity, as it is observed that the slope value is higher when compared with the Rayleigh mode. However, for the pseudo-bulk mode, the phase velocity showed an significant increase with the increase in wavelength, and did not appear to be directly proportional to the IDT size. It exhibits a larger phase velocity shift between IDT sizes. This due to the PB wave mode penetrating deeply into sapphire and the velocities of the sapphire substrate being higher than the velocity of the GaN thin film. As reported by Pedros et al., the transverse wave of GaN (C${}_{44}$) is 3941 m/s, while the transverse wave of sapphire (C${}_{4}4$) is 5938 m/s [18]. The understanding of different modes in a single IDT size is important to determine the desired mode to be used in the biosensor. Moreover, the extracted shear horizontal waves in this work are very beneficial for liquid environment sensor applications, such as biosensors, as low attenuation occurs when in contact with a fluid.

## 4. Conclusions

In summary, we demonstrated experimentally and theoretically the presence of surface guided modes (Rayleigh and Sezawa) in a GaN/sapphire substrate with the addition of a thin guiding layer made from gold. The GaN epitaxial layer grown by MOCVD allowed to propagate different acoustic modes with a slight decrease in velocity due to the mass loading effect. Based on the displacement field, we could observe the energy confinement of surface guided modes with most of the energy located in the GaN and the top guiding layer. The presence of a surface guided mode triggered by a Sezawa wave could be promising for the development of high-frequency SAW-based devices, particularly for sensing and actuating purposes for telecommunication and biosensing applications.

## Data Availability

Not applicable.
